# Supplementary material for: Atomistic Representation of Anomalies in the Failure Behaviour of Nanocrystalline Silicene
Source: Sci Rep. 2017 Nov 7;7:14629. doi: 10.1038/s41598-017-15146-6 (PMC5676956; doi:10.1038/s41598-017-15146-6)
Supplement: Supplementary file 1 — Supplimentary Information [file 41598_2017_15146_MOESM1_ESM.pdf]

Atomistic Representation of Anomalies in the Failure Behaviour of Nanocrystalline Silicene

Tawfiqur Rakib[1], Sourav Saha[1*], Mohammad Motalab[1], Satyajit Mojumder[1], Md Mahbubul Islam[2*]

[1]Department of Mechanical Engineering, Bangladesh University of Engineering and Technology, Dhaka-1000, Bangladesh.

[2]School of Materials Engineering, Purdue University, West Lafayette, IN-47907, USA

[*]ssaha09@me.buet.ac.bd, [*]islam3@purdue.edu

1. Calculation of Grain Size

The randomly oriented grain structures in nanocrystalline silicene (nc-silicene) are constructed by Voronoi Tessellation method. Since the grain centres and crystallographic orientations are seeded randomly, it is difficult to find out the exact grain size of a nanocrystalline material. Therefore, a method is adopted to find out the average grain size of nc-silicene which is previously used in the literature[1]. In our study, the nc-silicene sheet is square shaped. By assuming square geometry for an equivalent average grain in nc-silicene sheet, the grain size is defined by- $\sqrt{\frac{L^2}{N}}$, where L is the length of the side of the sheet and N is the number of grains in the plane.

2. Statistical Analysis of Uncertainty

Since the GBs are oriented randomly, the nc-silicene sheet of the same grain size may behave differently. So there are uncertainties associated with the random grain boundary distributions in nc-silicene. To quantify these uncertainties, statistical analysis is performed on 10 different samples, and the results are incorporated with error bars in the paper. To demonstrate the difference in the behaviour stemming from the randomness, the stress-strain graph of a nc-silicene sheet of 5 nm grain size with 3 nm length of crack is shown in Fig. S1.

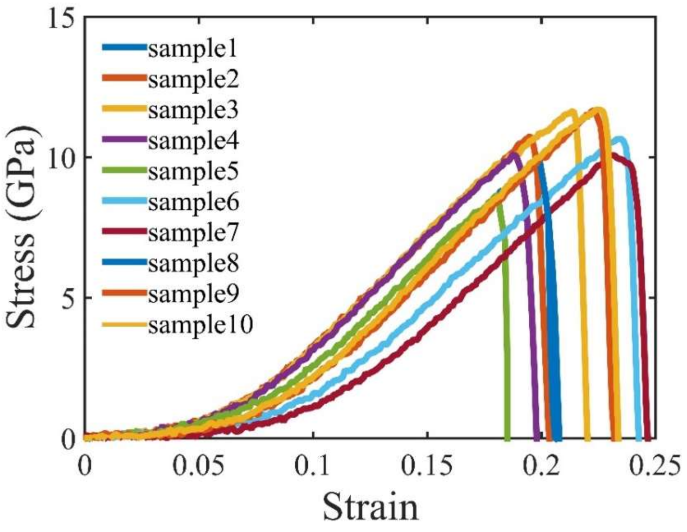

Figure S1: Stress-Strain graph of 10 random samples of a nc-silicene sheet of 5 nm grain size and 3 nm crack length.

3. Validation of the SW Potential Used

We performed a series of validation simulations to evaluate the performance of the SW potential used in this study in describing mechanical properties and fracture behaviour of nc-silicene. For this purpose, we used another optimised SW potential[2] and ReaxFF Si potential.[3] We denote the optimised SW potential used by us as "SW1" and the other SW potential as "SW2". All the three potentials are widely used for pristine silicene.

3.1 Geometric and Mechanical Properties

To validate the "SW1" potential for geometric and mechanical properties, we carried out conjugate gradient minimisation simulation and uniaxial tensile test on pristine silicene sheet of 30 nm × 30 nm at room temperature. After the minimisation, a buckled structure is obtained with a buckling height of h = 0.44 Å which is in good agreement with the previous literature[4]. The obtained Young's modulus is 85.3 GPa for the uniaxial tensile test. This value is in excellent agreement with the literature value of 82.2 GPa[5]. On the other hand, the buckling heights obtained by "SW2" and ReaxFF potential are 0.44 Å and 0.62 Å, respectively. The snapshot of the buckled structure of pristine silicene is shown below:

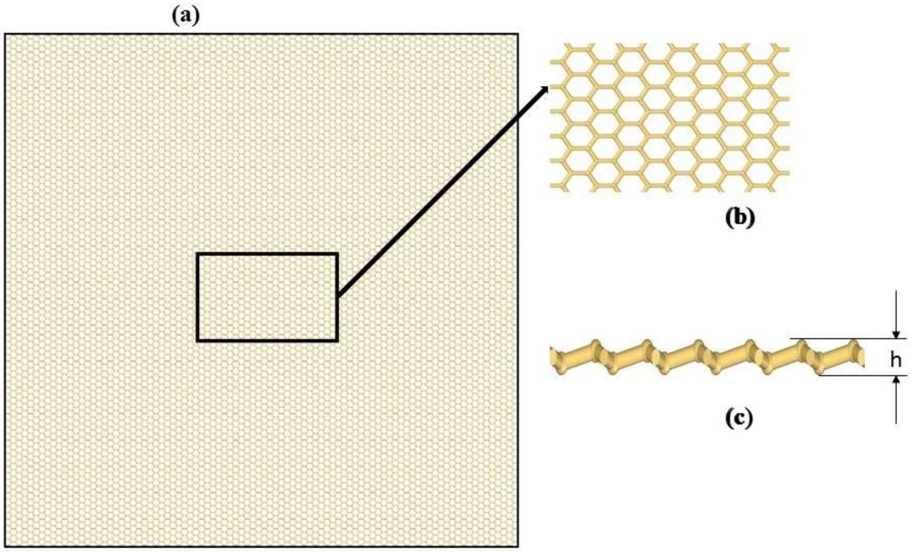

Figure S2: (a) A typical pristine silicene sheet, (b) zoomed view of it shows graphene-like hexagonal structure, and (c) the buckled structure of silicene obtained after minimisation.

3.2 Grain boundary energy of nc-silicene structures

Grain boundary energy is an important property for any polycrystalline material, which dictates the stability of the structures. Grain boundary (GB) energy is defined as the excess energy associated with the presence of grain boundaries with respect to the perfect structure. The grain boundary energies E_{GBE} for different grain sizes are calculated as[6]-

$$E_{GBE} = \frac{N(E_{GB} - E_{\mathrm{Pr}istine})}{2L_{GB}}, \tag{S1}$$

Where, N is the number of atoms of the system with grain boundary; E_{GB} and $\mathrm{E}_{\mathrm{Pristine}}$ are the per-atom energy of the system with GB and the pristine silicene sheets, respectively; L_{GB} is the length of the grain boundary of the nc-silicene. Here, the random GB distribution makes it difficult to find the length of the grain boundaries. Therefore, we used an image processing tool of MATLAB to find the GB length approximately.

The grain boundary energy of nc-silicene is calculated for three different potentials by using equation S1, and these are shown in the Fig. S3 as a function of grain size. It is found that the GB energy obtained by "SW1", "SW2", and ReaxFF potentials exhibit similar trend with varying grain sizes.

It is evident from Fig. S3 that nc-silicene sheet with an average grain size of 17.32 nm has the lowest GB energy, hence it is the most stable grain size. This is an intriguing fact that nc-silicene of grain size 17.32 nm is found to fail at the highest fracture stress in our study. One can also see that in Fig. S3, the GB energy is increasing for the grain sizes larger than 17.32 nm. On the other hand, for the grain sizes larger than 17.32 nm, the fracture stress decreases following the pseudo Hall-Petch relation. This minimum of GB energy in Fig. S3 corroborates our finding that there is a unique transition for fracture stress of nc-silicene from pseudo inverse Hall-Petch relation to inverse Hall-Petch relation at the grain size of 17.32 nm. From this analysis, it becomes evident that the nc-silicene sheet with lower GB energy has the higher fracture stress. Therefore, lower grain boundary energy indicates higher stability and higher fracture stress for nc-silicene. Previously, this type of behaviour was also observed for graphene[7]

where it is shown that armchair GB having higher GB energy than zigzag GB exhibits lower fracture stress.

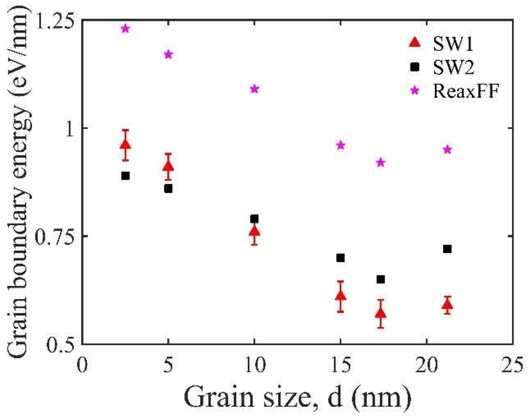

Figure S3: Variation of grain boundary energy with respect to grain size using "SW1", "SW2" and "ReaxFF" potentials. The values obtained from these three potentials follow the similar trend. For a grain size of 17.32 nm, our simulations predict the minimum grain boundary energy. **Since "SW1" potential is adopted in our study, the grain boundary energies were calculated from 10 different samples with random orientations to find statistical uncertainties.**

3.3 Vacancy formation energy

Vacancy formation energy of silicene is computed for monovacancy, bivacancy and Stone-Wales defect using "SW1", "SW2", and ReaxFF potentials. In a previous DFT study, monovacancy, divacancy and Stone-Wales defect formation energies were used to characterise the stability of the silicene system with defects[8]. Since grain boundaries are associated with defects, as such vacancy formation energy will dictate the stability of the nc-silicene system. Therefore, we explored the stability of silicene system with defects and validated our results with respect to three potentials and available literature. The vacancy formation energy E_f is determined as

$$E_f = E_T - NE_{\mathrm{Pr}istine}, \qquad \text{(S2)}$$

Where, E_T is the total energy of the silicene sheet with defects; N is the number of atoms of defective silicene and $E_{Pristine}$ is the per-atom energy of the pristine silicene. Using the Equation S2, the vacancy formation energy is computed and listed in Supplementary Table S1.

Table S1: Vacancy formation energy (in eV) of silicene with monovacancy, bivacancy and Stone-Wales defects

Type of defect	SW1	SW2	ReaxFF	*ab-initio*[8]
Monovacancy	2.65	2.63	3.34	3.01
Bivancancy	3.23	3.66	4.46	3.70
Stone Wales defect	1.77	1.78	1.85	2.09

Table S1 shows that for "SW1" potential, the errors are around 12%, 12.5% and 15%, for the monovacancy, bivacancy and Stone-Wales defect, respectively. In these cases, we can see the error margins are closer to each other for mono and bivacancies. In case of Stone-Wales defect, it is slightly higher. We know that Stone-Wales defect does not have any cluster of vacancies, rather this is due to an irregular bond pattern. Therefore, this might be a reason for this higher error margin. It is also known that DFT results inherently possess an error margin, therefore, a rigorous validation of these data can only be possible comparing with experiment, which has not yet been performed.

Although the SW1 potential predicted monovacancy, bivacancy, and Stone-Wales defects quite nicely with a reasonable error margin, it is imperative to evaluate its accuracy in describing crack propagation behaviour. Since cracks are essentially a cluster of vacancies, we extended our validation calculations up to clusters of six vacancies. The computed data are shown in Table S2 along with the DFT values[9] for 3-6 vacancies. The geometries and vacancy positions used in our calculations are similar to the configurations used in the literature[9].

The data presented in Table S2 indicates that the percentage of errors by "SW1" potential for these four vacancies are 13%, 13.5%, 10% and 9.5% respectively. Therefore, the error in the vacancy formation energies obtained by "SW1" potential compared to DFT results are not increasing with the increase in the number of vacancies, rather, it is showing a relatively consistent error margin within the acceptable range of 9%-15%. Which indicates the SW1 potential can reasonably describe the crack propagation behaviour in the nc-silicene.

Table S2: Vacancy formation energy (in eV) of silicene with 3-6 vacancies.

Type of defect	SW1	SW2	ReaxFF	*ab-initio*[9]
V_3 (5-10_1-5)	4.15	4.29	5.58	4.768
V_4 (5-12_2-5)	4.86	5.13	6.69	5.641
V_5 (5-11_1-5)	5.77	5.98	7.82	6.408
V_6 (5-5-10-5-5)	6.02	6.13	6.72	6.652

3.4 Edge energy

The edge energies of nc-silicene are calculated using all three potentials and listed in Supplementary Table S3, and one can see that the calculated values are in good agreement with each other.

Table S3: Edge energy of silicene in armchair and zigzag directions

Average grain size	Edge energy (eV/nm)		
	SW1	SW2	ReaxFF
Pristine (Armchair)	3.94	3.75	2.64
Pristine(Zigzag)	7.61	7.29	5.08

3.5 Grain Boundary Energy as a Function of Misorientation Angle

We further studied the grain boundary energy for different misorientation angles (0^0-60^0) of tilt grain boundary in nc-silicene by using "SW1" and ReaxFF potentials. For these calculations, we consider a sample of bi-crystal silicene with a symmetric tilt grain boundary. The structures are minimised using conjugate gradient minimisation scheme. The grain boundary energy is calculated using the equation S1. In this case, L_{GB} is taken as the length of the side along the tilt grain boundary direction. The results from both the potentials indicate a good agreement with each other. The grain boundary energy concerning the misorientation angle is shown in Figure S4. It is found that for low angles, the grain boundary energy is less, hence more stable. As we see, both the potentials predict lower minima at 40^0 and 60^0 misorientation angles. Because in the case of 40^0 and 60^0 misorientation angle, the defect rings

are accommodated with lower lattice mismatch. Therefore, they may lead to lower GB energies despite the high misorientation angle as lower the lattice mismatch, lower the GB energy[10]. The similar trend of lower GB energies at high angles was also found in graphene[11].

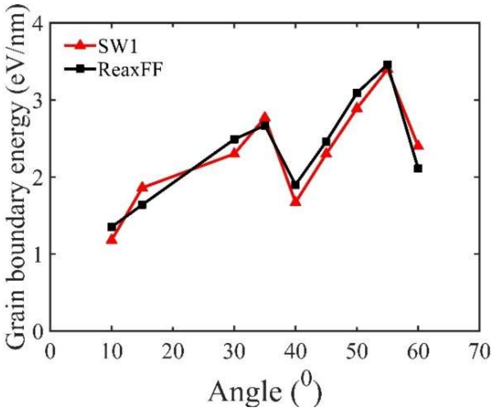

Figure S4: Variation of grain boundary energies with different misorientation angle by using "SW1" potential and ReaxFF potential. Minima for GB energy are found at 40^0 and 60^0 angles.

4. Effect of strain rate on crack sensitive and insensitive fracture

Strain rate has a significant influence on the material properties and is an important parameter for MD simulations for conserving energies. Yet, a high strain rate is generally adopted for the MD simulations due to the constraint in computational resources. Since strain rate can affect the fracture behaviour significantly, we explored the cases demonstrated in the paper by lower strain rates. In Supplementary Figs. S5 and S6, the atomic configurations of crack insensitive and sensitive fracture are shown before and after the fracture at strain rates of 7.5×10^8 s^{-1}, 5×10^8 s^{-1}, and 10^8 s^{-1}. At lower strain rates, it is found that the mechanism of crack insensitive fracture and the crack sensitive fracture is similar to that at a high strain rate of 10^9 s^{-1}.

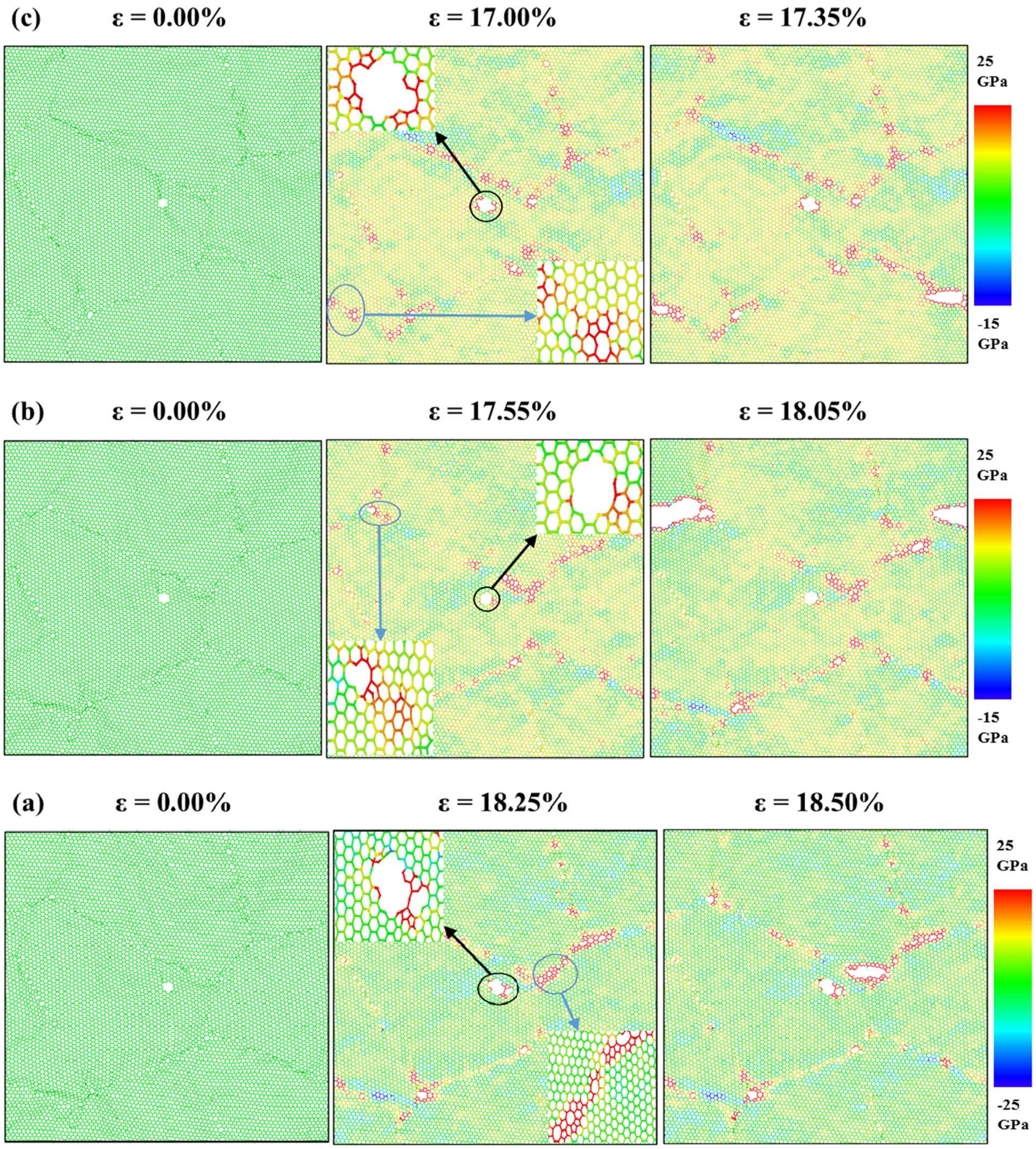

Figure S5: Atomistic configurations of crack insensitive fracture of nc-silicene 15 nm grain size with crack length 1 nm at strain rates (a) 7.5 × 10^8 s^{-1}, (b) 5 × 10^8 s^{-1}, and (c) 10^8 s^{-1}. The blue marked regions indicate the stress concentration region near the crack tip and the black marked regions indicate the stress concentration region along the GB. The "zoom-in" view of the marked region is also included.

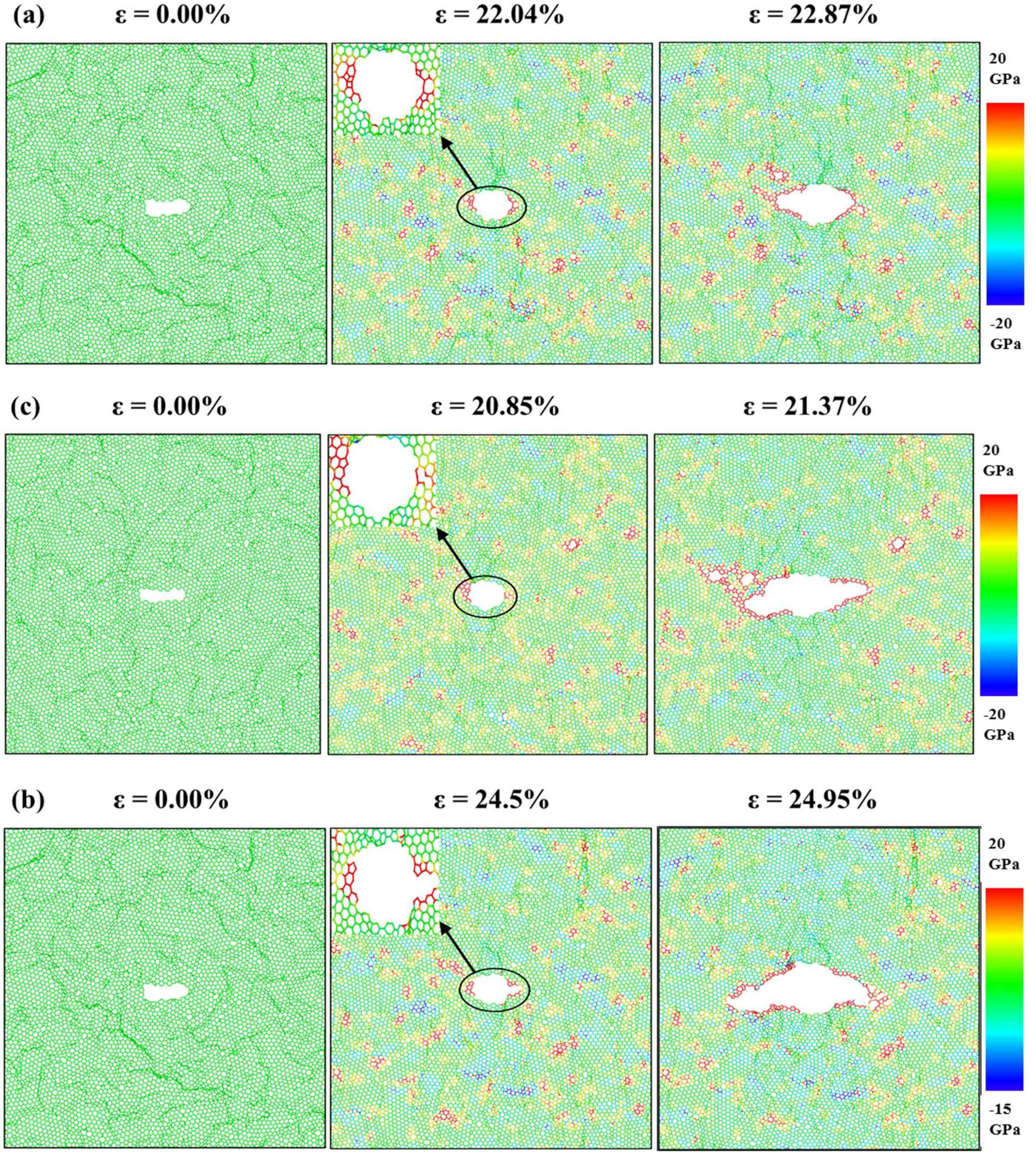

Figure S6: Atomistic configurations of crack sensitive fracture of nc-silicene of 2.5 nm grain size with 4 nm length of the crack at strain rates (a) 7.5 × 10^8 s^{-1}, (b) 5 × 10^8 s^{-1}, and (c) 10^8 s^{-1}. Here, the black marked regions indicate the stress concentration region near the crack. The "zoom-in" view of the marked region is also included.

The effects of the strain rate on the fracture stress and strain are demonstrated in Supplementary Fig. S7. However, we observed that lowering strain rate further decreases the fracture stress. However, the variation falls within the error bar of our calculations which affirms that the strain rate used in this study is reasonable to investigate the material response under tensile loading.

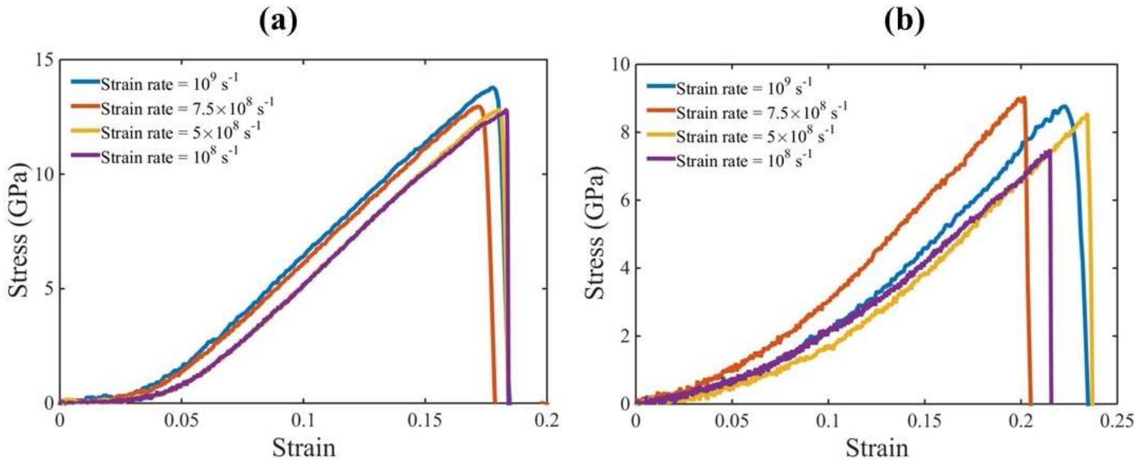

Figure S7: Stress-Strain graph at different strain rates for (a) crack insensitive fracture of nc-silicene of grain size 15 nm with crack length 1 nm and (b) crack sensitive fracture of nc-silicene of grain size 2.5 nm with crack length 4 nm.

Supplementary References

1. Mortazavi, B., Pötschke, M. & Cuniberti, G. Multiscale modeling of thermal conductivity of polycrystalline graphene sheets. *Nanoscale* **6,** 3344–3352 (2014).
2. Zhang, X. *et al.* Thermal conductivity of silicene calculated using an optimized Stillinger-Weber potential. *Phys. Rev. B* **89,** 54310 (2014).
3. Fogarty, J. C., Aktulga, H. M., Grama, A. Y., van Duin, A. C. T. & Pandit, S. A. A reactive molecular dynamics simulation of the silica-water interface. *J. Chem. Phys.* **132,** 174704 (2010).
4. Peng, Q., Wen, X. & De, S. Mechanical stabilities of silicene. *RSC Adv.* **3,** 13772–13781 (2013).
5. Pei, Q.-X., Sha, Z.-D., Zhang, Y.-Y. & Zhang, Y.-W. Effects of temperature and strain rate on the mechanical properties of silicene. *J. Appl. Phys.* **115,** 23519 (2014).

6. Liu, T.-H., Gajewski, G., Pao, C.-W. & Chang, C.-C. Structure, energy, and structural transformations of graphene grain boundaries from atomistic simulations. *Carbon* **49,** 2306–2317 (2011).
7. Cao, A. & Yuan, Y. Atomistic study on the strength of symmetric tilt grain boundaries in graphene. *Appl. Phys. Lett.* **100,** 211912 (2012).
8. Gao, J., Zhang, J., Liu, H., Zhang, Q. & Zhao, J. Structures, mobilities, electronic and magnetic properties of point defects in silicene. *Nanoscale* **5,** 9785–9792 (2013).
9. Li, S. *et al.* Defects in Silicene: Vacancy Clusters, Extended Line Defects, and Di-adatoms. *Sci. Rep.* **5,** (2015).
10. Zhang, J., Zhao, J. & Lu, J. Intrinsic Strength and Failure Behaviors of Graphene Grain Boundaries. *ACS Nano* **6,** 2704–2711 (2012).
11. Liu, Y. & Yakobson, B. I. Cones, Pringles, and Grain Boundary Landscapes in Graphene Topology. *Nano Lett.* **10,** 2178–2183 (2010).
